# The top 5 causes of death in China from 2000 to 2017

**DOI:** 10.1038/s41598-022-12256-8

**Published:** 2022-05-17

**Authors:** Haiyin Zou, Zheng Li, Xinjie Tian, Yongcheng Ren

**Affiliations:** 1 Institute of Health Data Management, Huanghuai University, Zhumadian, 463000 Henan People’s Republic of China; 2 Zhumadian Central Hospital, Zhumadian, 463003 People’s Republic of China; 3 Department of Health Statistics, College of Public Health, Zhengzhou University, Zhengzhou, 450001 People’s Republic of China

**Keywords:** Cardiovascular diseases, Epidemiology, Health policy

## Abstract

Limited information is available on the epidemiological characteristics of major causes of death in the last 18 years. In this study, we investigated the epidemiological characteristics of the top 5 causes of death in China from 2000 to 2017. Data were obtained from the 18-year reports of Ministry of Health and analyzed by Grid Search Method, Permutation test, and log-linear regression model. The top 5 consistent causes of death, malignant tumor, cerebrovascular disease, heart trouble, respiratory disease, trauma and toxicosis accounted for 82.6% in 2000, 86.49% in 2017 in urban areas and 83.31% in 2000, 88.34% in 2017 in rural areas. The increasing trends (*P* < 0.05) of proportions of death of malignant tumor, cerebrovascular disease, and heart trouble have average annual percent change (AAPC) = 0.5%, 0.3%, 2.4% in urban areas and 1.7%, 1.5%, 4.3% in rural areas. The AAPCs of respiratory disease are − 1.4% in urban areas and − 3.6% in rural areas. Cardio-cerebrovascular disease increased (Urban: 39.02% to 43.56%, AAPC = 1.3%, *P* < 0.05; Rural: 32.03% to 45.91%, AAPC = 2.7%, *P* < 0.05) steeply from 2000 to 2017 which are higher than that of malignant tumor (*P* < 0.05). The top 5 causes of death in China accounted for more than 85% of all deaths in 2017, in which cardio-cerebrovascular disease accounted for the largest proportion with the steepest increasing trend.

## Introduction

One of the most important ways to assess the effectiveness of a country's health system is to measure the number of deaths and their causes each year^1^. It is also important to understand the way how diseases and injuries affect people. Health authorities could use the statistics data of different causes of death during different periods as an effective tool to establish their focus in public heath area which may help both to extend the lifespan and to improve the life quality of their people^1^.

The noncommunicable diseases caused by aging and health transitions have brought huge burden to the developing countries, increasing the most economically productive age span rapidly in those countries. Therefore, to developing countries, adult mortality rate becomes an important indicator for a comprehensive assessment of the mortality pattern in the population^2^. It is critical to improve the quality of cause-of-death data to improve the health conditions and reduce preventable deaths in population of the developing countries. In 1987, Ministry of Health of China established a vital registration system to record the incidence and causes of death. Until 2005, this system had covered 41 urban sites and 85 rural sites with population from 30,000–70,000^3,4^. From 2006 to the present, the cause-of-death monitoring system which includes 161 monitoring sites, has been carried out on the National Disease Surveillance System^4^.

From 2000 to 2017, there was an acceleration of urbanization and an economic boom in China. Risk factors for death have shifting patterns, and population health have fundamental transformations. However, few studies in China have revealed these transition trends, leaving the relations between population health and the development of society poorly characterized. Such information is crucial for population health professionals and policymakers to determine the next step in public health work and political reform in China. Our study analyzed the nationally representative data provided by the Ministry of Health and explored the epidemiological characteristics and secular trends of the top 5 causes of death in China from 2000 to 2017, with a close examination of demographic and ecological changes, trying to find the focus of intervention to reduce the risk of death.

## Methods

### Study design and sample

In this study, data of the proportion and causes of death were obtained from the China Statistical Yearbooks (2001–2018)^5^, which have been provided by the Ministry of Health and published by national bureau of statistics of China annually since 2001. The original sample was collected by using multistage stratified and cluster probability proportional sampling methods, which consists of participants from the general population of China with the provincial units as a secondary population, but excludes the population of Hong Kong, Macao and Taiwan. Data of diseases were classified based on the International Classification of Diseases (ICD-10). The specific ICD-10 code varies for each aggregated cause of death which follows the description in the Disease Classification and Code (GB/T14396-2001), issued by the Ministry of Health.

### Statistical analysis

Grid Search Method and Permutation test were used to find the joinpoints^6^. The log-linear regression model was used to determine the statistically significant trend change connection points in the model through Permutation test, limiting at most one trend point. Annual Percent change (APC) and average annual Percent change (AAPC) in each time period were calculated. If AAPC is completely located in a single junction segment, then AAPC = APC, indicating that the data of this group generally shows a monotonic upward or downward trend. All data are expressed as percentages. The 95% confidence interval (95% CI) of APC was estimated by using the T-distribution, and the AAPC confidence interval was estimated by using the normal distribution. If AAPC was located within a single junction segment, it was estimated using the T-distribution. Statistical analysis was performed using Joinpoint Regression Program 4.7.0.0 (National Cancer Institute, NCI, National Center for Statistical Research and Application of USA) and Excel 365 software. *P* < 0.05 (two-sided) is considered statistically significant.

### Ethical approval

This article does not contain any studies with human participants or animals performed by any of the authors.

## Results

The top 5 consistent causes of death are malignant tumor, cerebrovascular disease, heart trouble, respiratory disease, and trauma and toxicosis, of the period from 2000 to 2017. These 5 diseases account for 82.6% in 2000, 86.49% in 2017 in urban areas and 83.31% in 2000, 88.34% in 2017 in rural areas of the total deaths, respectively. The corresponding data were 83.99%, 87.15%, 83.40%, and 88.93% in males and 80.90%, 85.59%, 81.63%, and 87.51% in females. In urban areas, the first cause of death is malignant tumor continuously, while cerebrovascular disease and heart trouble flip in the second and third places, from 2000 to 2017. In 2000 and 2001, the first cause of death in rural areas, is respiratory disease, which drops to the third in 2002, and has dropped to the fourth by 2009, being replaced by heart trouble. After 2009, the top 3 consistent causes of death in rural areas are malignant tumor, cerebrovascular disease and heart trouble. Trauma and toxicosis is the fifth top cause of deaths in both females and males continuously, and it accounts for a higher proportion of death in males than in females (*P* < 0.05), especially in rural areas.

In urban areas (Table 1, Online Supplementary Figure s1), trend analysis based on joinpoint regression program shows that the AAPC of malignant tumor, heart trouble, and respiratory disease are 0.5%, 2.4%, and − 1.4% from 2000 to 2017, respectively (*P* < 0.05). The proportion of deaths caused by malignant tumor has an increasing trend from 2000 to 2008, and its APC = 1.7% (*P* < 0.05) (Online Supplementary Figure s1-A3). However, the trend is not significant from 2008 to 2017 (*P* > 0.05). The proportions of deaths caused by cerebrovascular disease and heart trouble increase with APC = 0.7% and APC = 2.9% (Online Supplementary Figure s1-B3, C3), respectively (*P* < 0.05), from 2002 to 2017. Stratification analysis by gender reveals that the proportion of deaths caused by trauma and toxicosis from 2000 to 2017 has a declining trend with AAPC =  − 1.1% (*P* < 0.05) in females, while the trend is not significant in males.

**Table 1:** The trends of the top 5 causes of death in urban areas of China from 2000 to 2017.

Gender	Diseases	Trends (1)		Trends (2)		AAPC (95% CI, %)
		Year-range	APC (95% CI, %)	Year-range	APC (95% CI, %)	
Male	Malignant Tumor	2000–2007	**1.8 (0.2**–3.4)*	2007–2017	− 0.5 (− 1.4–0.4)	0.4 (− 0.1–0.9)
	Cerebrovascular Disease	2000–2002	− 8.4 (− 21.7–7.2)	2002–2017	**0.8 (0.1**–1.5)*	0.3 (− 0.3–1.0)
	Heart Trouble	2000–2002	− 7.1 (− 25.2–15.3)	2002–2017	**2.7 (1.8**–3.6)*	**2.2 (1.4**–3.0)*
	Respiratory Disease	2000–2010	− **1.9 (**− **3.7**– − 0.1)*	2010–2017	0.1 (− 3.0–3.3)	− **1.1 (**− **1.9**– − 0.4)*
	Trauma and Toxicosis	2000–2017	− 0.5 (− 1.7–0.7)	–	–	− 0.5 (− 1.7–0.7)
Female	Malignant Tumor	2000–2017	0.7 (0.1–1.3)*	–	–	0.7 (0.1–1.3)*
	Cerebrovascular Disease	2000–2002	− 5.7 (− 17–7.2)	2002–2017	**0.8 (0.2**–1.3)*	0.4 (− 0.1–0.9)
	Heart Trouble	2000–2002	− 6.7 (− 28.9–22.4)	2002–2017	**3.2 (2.1**–4.4)*	**2.7 (1.8**–3.6)*
	Respiratory Disease	2000–2009	− **3.8 (**− **6.1**– − 1.5)*	2009–2017	0.3 (− 2.5–3.2)	− **1.9 (**− **2.9**– − 1.0)*
	Trauma and Toxicosis	2000–2002	9.4 (− 20.8–51.0)	2002–2017	− **1.6 (**− **2.9**– − 0.3)*	− 1.1 (− 2.3–0.2)
All	Malignant Tumor	2000–2008	**1.7 (0.1** − 3.4)*	2008–2017	− 0.5 (− 1.8–0.9)	**0.5 (0.1**–1.0)*
	Cerebrovascular Disease	2000–2002	− 6.6 (− 19.0–7.7)	2002–2017	**0.7 (0.1**–1.3)*	0.3 (− 0.2–0.9)
	Heart Trouble	2000–2002	− 6.7 (− 24.3–15)	2002–2017	**2.9 (2.0**–3.8)*	**2.4 (1.6**–3.1)*
	Respiratory Disease	2000–2010	− **2.4 (**− **4.1**– − 0.7)*	2010–2017	0.2 (− 2.8–3.3)	− **1.4 (**− **2.2**– − 0.7)*
	Trauma and Toxicosis	2000–2002	8.2 (− 21.6–49.3)	2002–2017	− 1.2 (− 2.5–0.2)	− 0.7 (− 1.9–0.5)

*APC* Annual Percent Change; *AAPC* Average Annual Percent Change; *CI* confidence interval.

Significant values are in bold.

*Indicates that AAPC or APC is significantly different from zero at the alpha = 0.05 level.

In rural areas (Table 2, Online Supplementary Figure s1), the trends of the proportions of the top 5 causes of death are all significant except trauma and toxicosis in overall from 2000 to 2017. The AAPC of malignant tumor, cerebrovascular disease, heart trouble, and respiratory disease are 1.7%, 1.5%, 4.3%, and − 3.6%, respectively (*P* < 0.05). Stratification analysis by gender reveals that the AAPC of the proportion of deaths caused by respiratory disease from 2000 to 2017, lies entirely within a single joinpoint segment, with a declining trend and AAPC =  − 3.3% in males (Online Supplementary Figure s2-D1), AAPC =  − 4.0% in females (Online Supplementary Figure s2-D2), respectively (*P* < 0.05). Meanwhile, the declining trend of the proportions of deaths of trauma and toxicosis is significant only in females with AAPC =  − 2.5% (*P* < 0.05).

**Table 2:** The trends of the top 5 causes of death in rural areas of China from 2000 to 2017.

Gender	Diseases	Trends (1)		Trends (2)		AAPC (95% CI, %)
		Year-range	APC (95% CI, %)	Year-range	APC (95% CI, %)	
Male	Malignant Tumor	2000–2007	**4.5 (2.5**–6.6)*	2007–2017	− 1.1 (− 2.2–0.1)	**1.0 (0.2**–1.8)*
	Cerebrovascular Disease	2000–2017	**1.4 (0.7**–2.2)*	–	–	**1.4 (0.7**–2.2)*
	Heart Trouble	2000–2005	0.1 (− 5.3–5.8)	2005–2017	**5.0 (3.5**–6.6)*	**3.9 (2.9**–4.9)*
	Respiratory Disease	2000–2017	− **3.3 (**− **4.5**– − 2.2)*	–	–	− **3.3 (**− **4.5**– − 2.2)*
	Trauma and Toxicosis	2000–2017	− 0.6 (− 1.6–0.4)	–	–	− 0.6 (− 1.6–0.4)
Female	Malignant Tumor	2000–2017	1.0 (− 0.1–2.0)	–	–	1.0 (− 0.1–2.0)
	Cerebrovascular Disease	2000–2003	**10.7 (0.9**–21.4)*	2003–2017	**1.2 (0.3**–2.1)*	**2.8 (1.2**–4.5)*
	Heart Trouble	2000–2005	0.7 (− 4.4–6.1)	2005–2017	**5.1 (3.7**–6.6)*	**4.1 (3.2**–5.0)*
	Respiratory Disease	2000–2017	− **4.0 (**− **5.3**– − 2.8)*	–	–	− **4.0 (**− **5.3**– − 2.8)*
	Trauma and Toxicosis	2000–2003	− **13.7 (**− **22.9**– − 3.4)*	2003–2017	0.1 (− 1.0–1.1)	− **2.5 (**− **4.4**– − 0.6)*
All	Malignant Tumor	2000–2003	**11.4 (1.8**–21.9)*	2003–2017	− 0.3 (− 1.1–0.6)	**1.7 (0.1**–3.3)*
	Cerebrovascular Disease	2000–2017	**1.5 (0.6**–2.5)*	–	–	**1.5 (0.6**–2.5)*
	Heart Trouble	2000–2005	0.1 (− 4.3–4.7)	2005–2017	**5.5 (4.3**–6.8)*	**4.3 (3.4**–5.2)*
	Respiratory Disease	2000–2017	− **3.6 (**− **4.8**– − 2.3)*	–	–	− **3.6 (**− **4.8**– − 2.3)*
	Trauma and Toxicosis	2000–2003	− **13.2 (**− **23.1**– − 2.1)*	2003–2017	0.8 (− 0.3–1.9)	− 0.7 (− 2.1–0.8)

*APC* Annual Percent Change; *AAPC* Average Annual Percent Change; *CI* confidence interval.

Significant values are in bold.

*Indicates that AAPC or APC is significantly different from zero at the alpha = 0.05 level.

**Figure 1:**
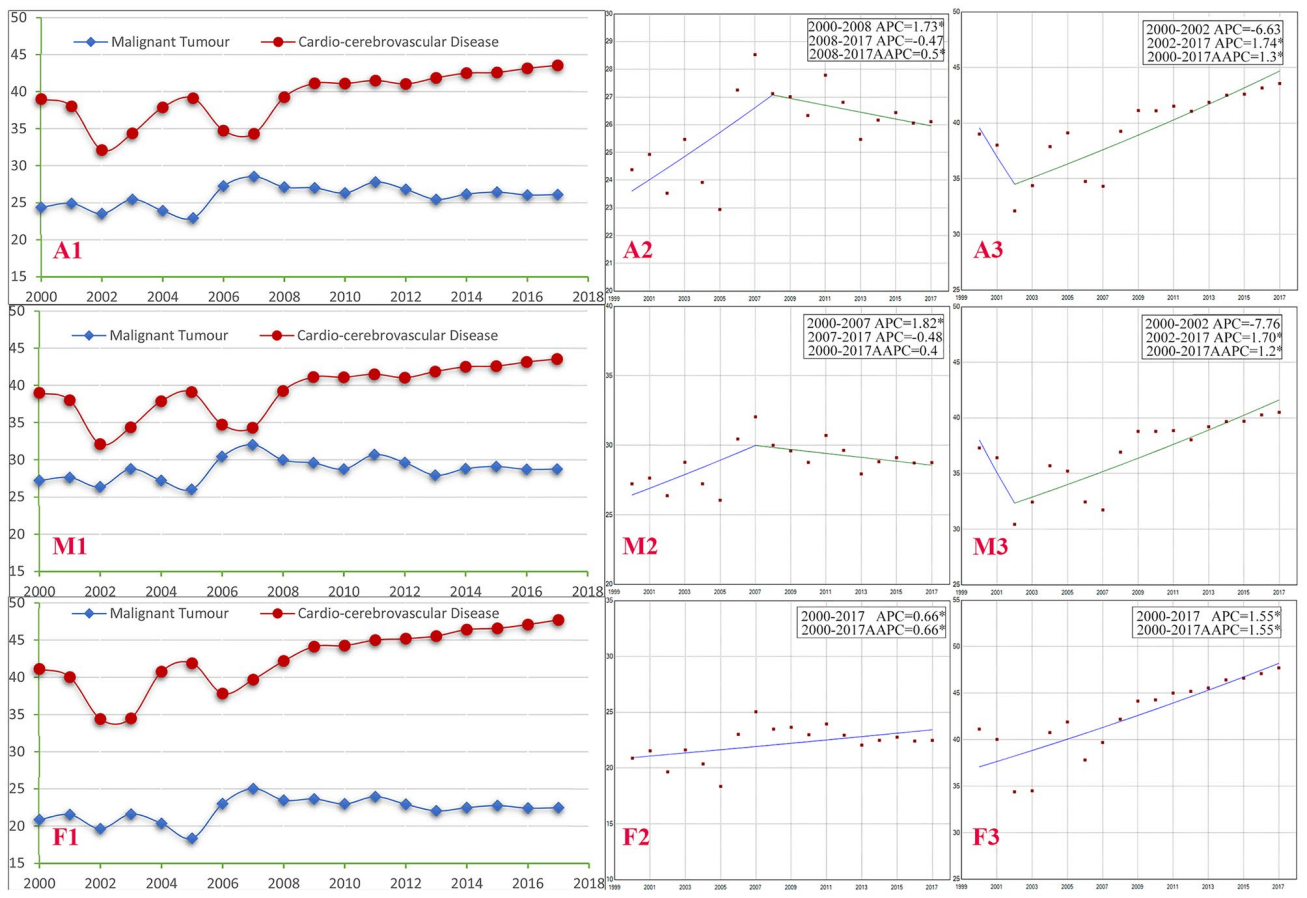
Comparison of trends for malignant tumor and cardio-cerebrovascular disease as causes of death in urban areas of China from 2000 to 2017. *Note A* all; *M* male; *F* female; *1* line chart based on raw data; *2* Joinpoints graph for malignant tumor; *3* Joinpoints graph for cardio-cerebrovascular disease; *APC* Annual Percent Change; *AAPC* Average Annual Percent Change; *Indicates that AAPC or APC is significantly different from zero at the alpha = 0.05 level. x-axis: year; y-axis: the proportion of deaths.

**Figure 2:**
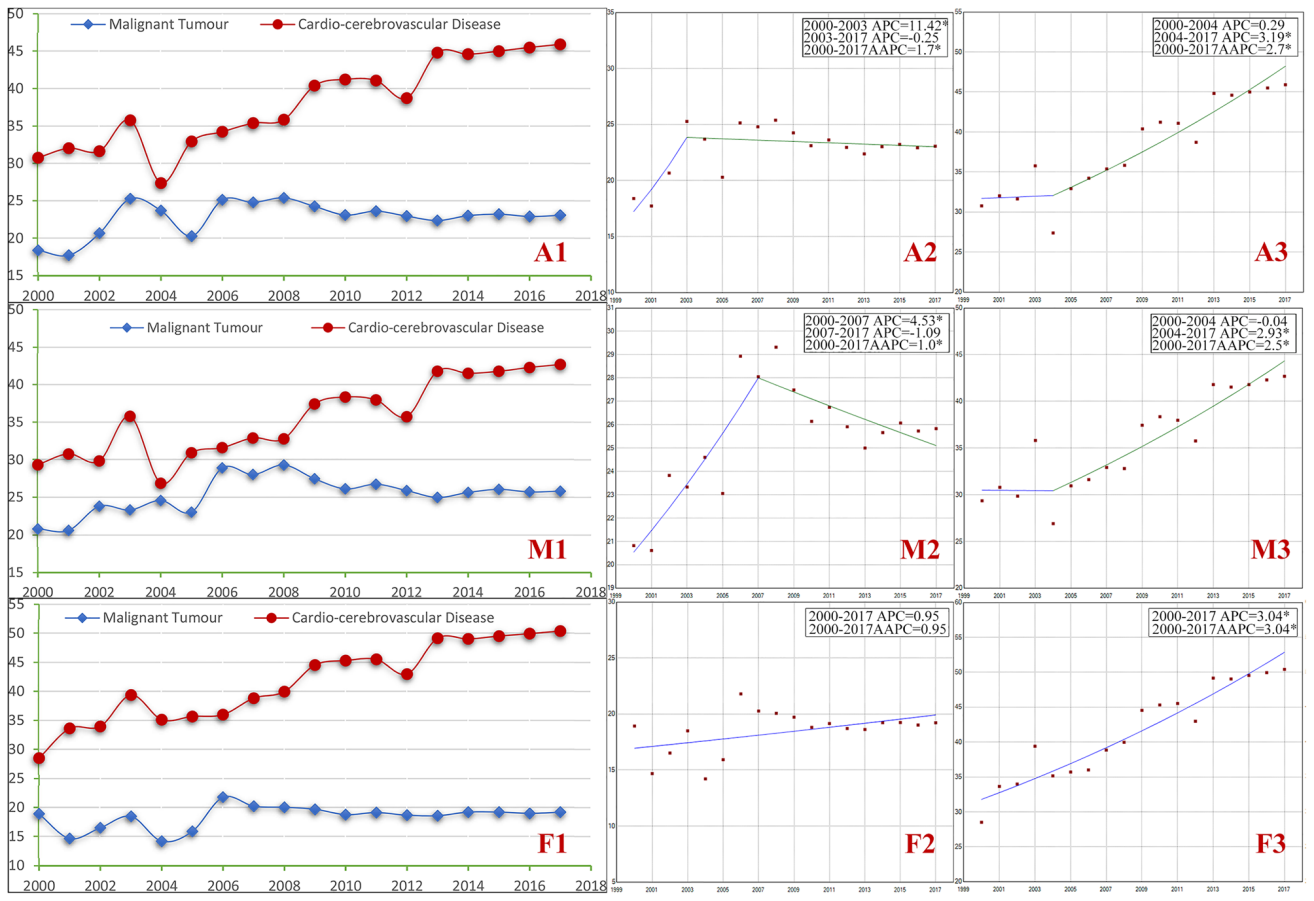
Comparison of trends for malignant tumor and cardio-cerebrovascular disease as causes of death in rural areas of China from 2000 to 2017. *Note A* all; *M* male; *F* female; *1* line chart based on raw data; *2* Joinpoints graph for malignant tumor; *3* Joinpoints graph for cardio-cerebrovascular disease; *APC* Annual Percent Change; *AAPC* Average Annual Percent Change; *Indicates that AAPC or APC is significantly different from zero at the alpha = 0.05 level. x-axis: year; y-axis: the proportion of deaths.

The combined proportion of deaths due to cerebrovascular disease and heart trouble increases (39.02% to 43.56%, *P* < 0.05) from 2000 to 2017, which is higher than that due to malignant tumor in overall in urban areas (*P* < 0.05) (Fig. 1-A1). The AAPC of the proportion of deaths of cardio-cerebrovascular disease from 2000 to 2017 lies within 2 joinpoint segments (AAPC = 1.3%, *P* < 0.05), with an increasing trend from 2002 to 2017 and APC = 1.74% (*P* < 0.05), (Fig. 1-A3), which is higher than that of malignant tumor (Fig. 1). The results of stratification analysis by gender is similar. In rural areas, the proportion of deaths of cardio-cerebrovascular disease increases (32.03% to 45.91%, *P* < 0.05) from 2000 to 2017 and is higher than that of malignant tumor in overall (*P* < 0.05) (Fig. 2-A1). The AAPC of the proportion of deaths of cardio-cerebrovascular disease is 2.7% (*P* < 0.05) (Fig. 2-A3), with a peak in females at 3.04% (*P* < 0.05) (Fig. 2-F3). Overall, the increasing trend of the proportions of deaths of cardio-cerebrovascular disease is steeper than that of malignant tumor, which is not related to gender (Figs. 1, 2).

## Discussion

This study reveals that in China, the top 5 consistent causes of death are malignant tumor, cerebrovascular disease, heart trouble, respiratory disease, and trauma and toxicosis during the past 18 years. All these 5 causes account for 82.6% in 2000, 86.49% in 2017 in urban areas and 83.31% in 2000, 88.34% in 2017 in rural areas of the total deaths. Trend analysis results suggest that the proportions of death of malignant tumor, cerebrovascular disease, and heart trouble have significantly increased, while the one of respiratory disease has significantly decreased. The declining trend of the proportion of death of trauma and toxicosis is significant only in females. In addition, the increasing trend of the proportion of death of cardio-cerebrovascular disease is steeper than that of malignant tumor (AAPC: Urban, 1.5% vs 0.5%; Rural, 2.7% vs 1.7%), the decreasing trend of the proportion of respiratory disease in rural areas is steeper than that in urban areas (AAPC: − 3.6% vs − 1.4%).

Malignant tumor is the first-leading cause of death in the world. The GLOBOCAN 2018 estimated that there were 18.1 million new cases of malignant tumor and 9.6 million related deaths in 2018^7^. The large burden of malignant tumor is projected to increase with a predicted 22 million new cases and 13 million related deaths occurring by 2030^8^. Previous studies have identified that the malignant tumor burden is greater in higher Human Development Index countries^9,10^, while a greater proportion of the global mortality burden was observed in low and medium Human Development Index countries. The proportion of malignant tumor incidence will experience a 100% and 81% increase in low and medium Human Development Index countries from 2008 to 2030, respectively^11^. Our study estimated that malignant tumor was the first-leading cause of death in China with AAPC = 0.5% and 1.7% from 2000 to 2017 in urban and rural areas, respectively. Malignant tumor-related deaths were more popular in urban than in rural areas. Even the proportion of death caused by malignant tumor presented a continuing upward trend during the past 18 years, the one in urban areas only continuously increased from 2000 to 2008. In urban areas, malignant tumor-related deaths accounted for 27.12% of total deaths in 2008, and its proportion has been fluctuating at a high level from 2008 to 2017. In rural areas, even malignant tumor-related deaths only accounted for 23.07% (lower than 25.50%) of total deaths in 2017, it presented a continuing upward trend from 2008 to 2017. Based on this result, we urgently need to control its occurrence in rural areas in China, but at the same time, we should also strengthen interventions for urban populations because of their high base.

In 2016, it was reported that CVD accounted for more deaths than tumor or any other disease did in China, which was the first-leading cause of death in China^12^. In 2018, National Center for Cardiovascular Diseases estimated that about 290 million patients were suffered from CVD in China, including 13 million from stroke, 11 million from coronary heart disease, 5 million from pulmonary heart disease, 4.5 million from heart failure, 2.5 million from rheumatic heart disease, 2 million from congenital heart disease and 245 million from hypertension^12^. Our study indicated that the proportion of death caused by malignant tumor was higher than that caused by cerebrovascular disease and heart trouble, but lower than that caused by CVD. CVD-related deaths accounts for more than 40% of total deaths after 2010, especially in females (more than 45%), with a peak of 50.4% in rural females in 2017. Increasing aging, lifestyle changes, psychological stress, and other unhealthy behaviors have led to a continuous increase in the number of heart trouble^13^, increasing the burden of cardiovascular disease, especially the increase in cardiovascular mortality in rural areas in China. Note that the proportion of death of heart trouble is rising fastest, especially in rural areas with AAPC = 4.3%. However, the density of health-care professionals is lower in rural than urban areas in China^14^. So, strengthening the prevention and treatment of CVD under the leadership of the government is an urgent task, both in urban and rural areas.

According to the Global Burden of Disease 2019 (GBD 2019) study and WHO, a total number of lives lost, is associated with three broad topics: cardiovascular, respiratory, and neonatal conditions. Respiratory diseases, especially respiratory infections, and tuberculosis; are closely related to economic development. People living in a low-income country are far more likely to die of respiratory diseases. With the development of the economy and the acceleration of urbanization in China, the proportion of death caused by respiratory diseases is decreasing, especially in rural areas with AAPC =  − 3.6% from 2000 to 2017. Moreover, respiratory diseases are also closely related to air quality^15^. China has experienced a severe deterioration in air quality over the past several years. However, air pollution control policies in China have been experiencing significant positive changes from 2013 to 2020, which might explain the reason why respiratory diseases have been controlled to be the fourth leading cause of death and showing a downward trend in China. Trauma and toxicosis is a significant public health problem and among the leading causes of death worldwide^16^. It was consistently the fifth leading cause of death in China from 2000 to 2017. Road traffic crashes^16^ and alcohol use^17^ are the leading risk factors for death and disability, particularly among males in China^18^, which contribute to health loss from many causes and exacted their toll throughout the rest of victims’ lifetime. Fall injury is a serious cause of morbidity among older people and its occurrence increases dramatically with age^19^. China has begun the accelerated aging process. As a result, there will be more and more older people in the future, and fall injury will be an important cause of trauma and toxicosis-related death. Therefore, in order to prevent trauma and toxicosis, these interventions such as speed enforcement and drink-driving enforcement need to be strengthened^20^.

Our study provides a chance demographically and epidemiologically for careful review of population health indicators at the national levels, which is critical for healthcare providers in China to develop evidence-based policies. The result of this study reflects both similarities and differences when compared with data from leading causes of death in the US from the years 2000 to 2017^21^. The top 5 causes of death in both China and the US include cancer, cardiovascular disease, cerebrovascular disease. Although these top causes of death remain the same in both China and the US, the trends of those disease mortalities in China are opposite to the ones in the US. Our data show that the mortalities of cardio-cerebrovascular disease and cancer in China have been significantly increasing. However, in the US, cardio-cerebrovascular disease mortality has been steadily declining from 1999 to 2017, with changes for heart disease mortality per 100,000 from 266.5 in 1999 to 165.0 in 2017, for stroke mortality from 61.6 to 37.6^22^ , and the cancer death rate has declined by 29% from 1991 to 2017, with an average of 1.5% per year from 2008 to 2017^23^.

The most important step in explaining the reason why the trends of death caused by cardio-cerebrovascular disease and cancer in China and the US are opposite is the examination of contributors to those mortality trends in each country. Ford et al. used IMPACT Coronary Heart Disease Model to examine contributors to the decline in coronary disease mortality rate in the US from 1980 to 2000, which shows the decreasing coronary disease mortality rate is likely attributable to a variety of factors, including improved primary health care and clinical management, changes in risk factors, and the relative contribution of primary and secondary prevention24. Ford et al. reported “secondary preventive therapies after myocardial infarction or revascularization (11%), initial treatments for acute myocardial infarction or unstable angina (10%), treatments for heart failure (9%), revascularization for chronic angina (5%), and other therapies (12%)”^24^, and “approximately 44% was attributed to changes in risk factors, including reductions in total cholesterol (24%), systolic blood pressure (20%), smoking prevalence (12%), and physical inactivity (5%)”^24^. In contrast, the increasing trends in the mortality rates of cardio-cerebrovascular disease and cancer in China are linked to an increased range of risk factors, including lack of evidence-based health education, unhealthy lifestyle choices, such as smoking, unhealthy diet, physical inactivity, and changes in social pressures and norms. To some extent, many of those risk factors are preventable, and some are avoidable under certain control^25,26^. Therefore, the examinations of contributors to the shifting patterns of leading causes of death provide insight into the direct and indirect effects of primary health care and secondary preventive therapies on mortality.

There might be several limitations in our study. First, our results are based on a secondary analysis of China Statistical Yearbook data although the data we used came from a wide range of years from 2000 to 2017. Due to the limitations of source data, we cannot obtain data for the overall country, and the reprocessing of data cannot be further adjusted nor standardized in this study. Second, we did not analyze the determinants of the causes of death since our study is an exploratory ecological trend study. Third, the results of our study can only give the weight of different causes of death and reflect the trend of proportion since this study is based on the composition of the causes of death rather than mortality.

In conclusion, the top 5 causes of death in China account for more than 85% of all deaths in 2017. The trends of all diseases have been increasing, except for respiratory disease. Cardio-cerebrovascular disease should be the primary focus for disease prevention and control since it has accounted for the largest proportion with the steepest increasing trend during the past 18 years.

## Supplementary Information

Supplementary Information 1.

Supplementary Information 2.

## Data Availability

The data that support the findings of this study are openly available in National Bureau of Statistics at http://www.stats.gov.cn/tjsj/ndsj/.
