# Supplementary material for: The top 5 causes of death in China from 2000 to 2017
Source: Sci Rep. 2022 May 17;12:8119. doi: 10.1038/s41598-022-12256-8 (PMC9114369; doi:10.1038/s41598-022-12256-8)
Supplement: Supplementary file 2 — Supplementary Information 2. [file 41598_2022_12256_MOESM2_ESM.pdf]

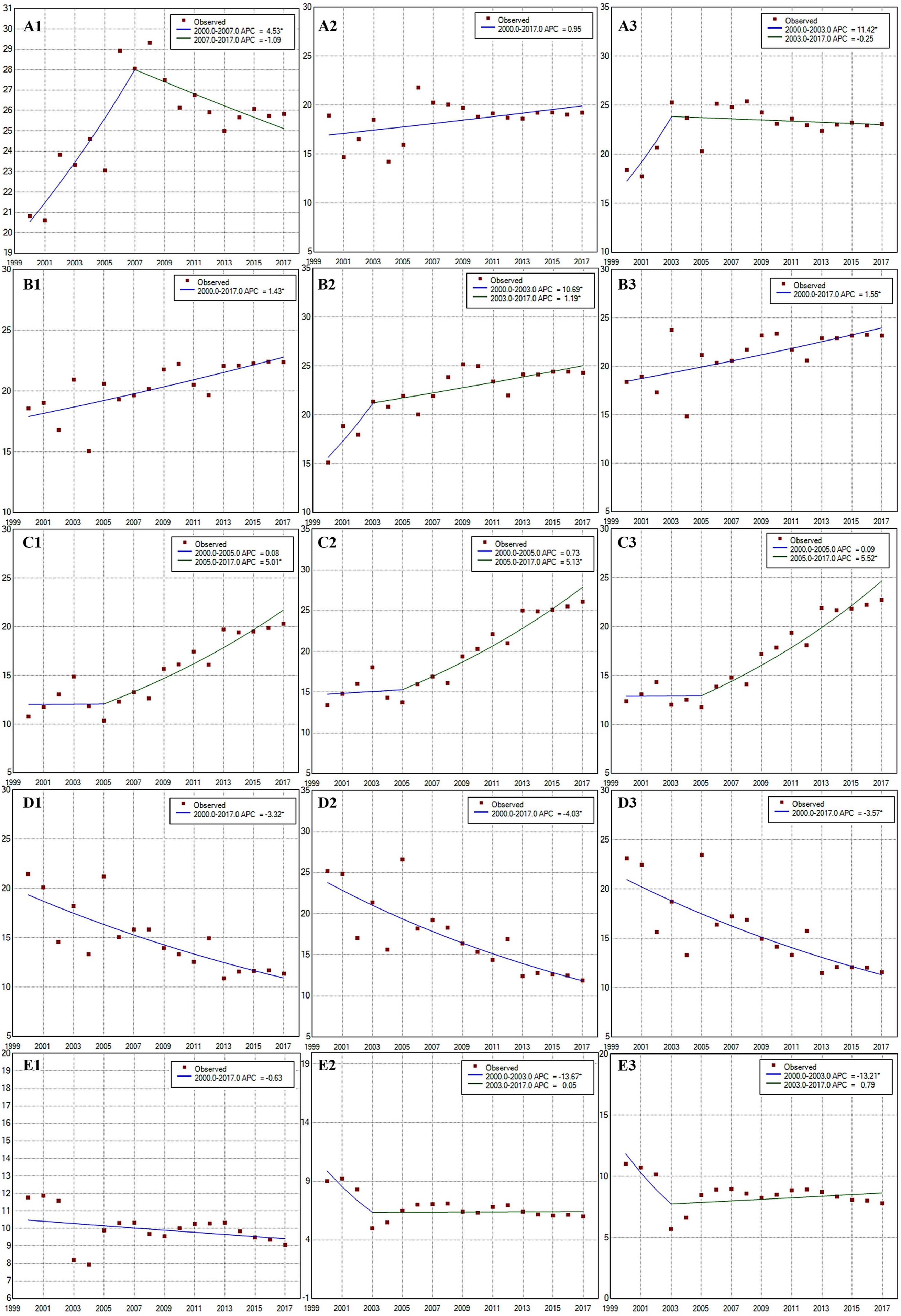

Online Supplementary Figure s2. The trends of the top 5 causes of death in rural areas of China from 2000 to 2017.

Note: A: Malignant Tumor; B: Cerebrovascular Disease; C: Heart Trouble; D: Respiratory Disease; E: Trauma and Toxicosis; 1: Male; 2: Female; 3: All; APC: Annual Percent Change; *: indicates that APC is significantly different from zero at the alpha=0.05 level.

x-axis: year; y-axis: the proportion of deaths.
